# Supplementary material for: Effects of Trichoderma harzianum combined with Phanerochaete chrysosporium on lignin degradation and humification during chicken manure and rice husk composting
Source: Front Microbiol. 2025 Feb 28;16:1515931. doi: 10.3389/fmicb.2025.1515931 (PMC11906335; doi:10.3389/fmicb.2025.1515931)
Supplement: Supplementary file 1 [file Data_Sheet_1.zip › 6.Function/CKS_HS/FunGuild/l2/metastats/Plant_Pathogen.HS_CKS.metastats.pdf]

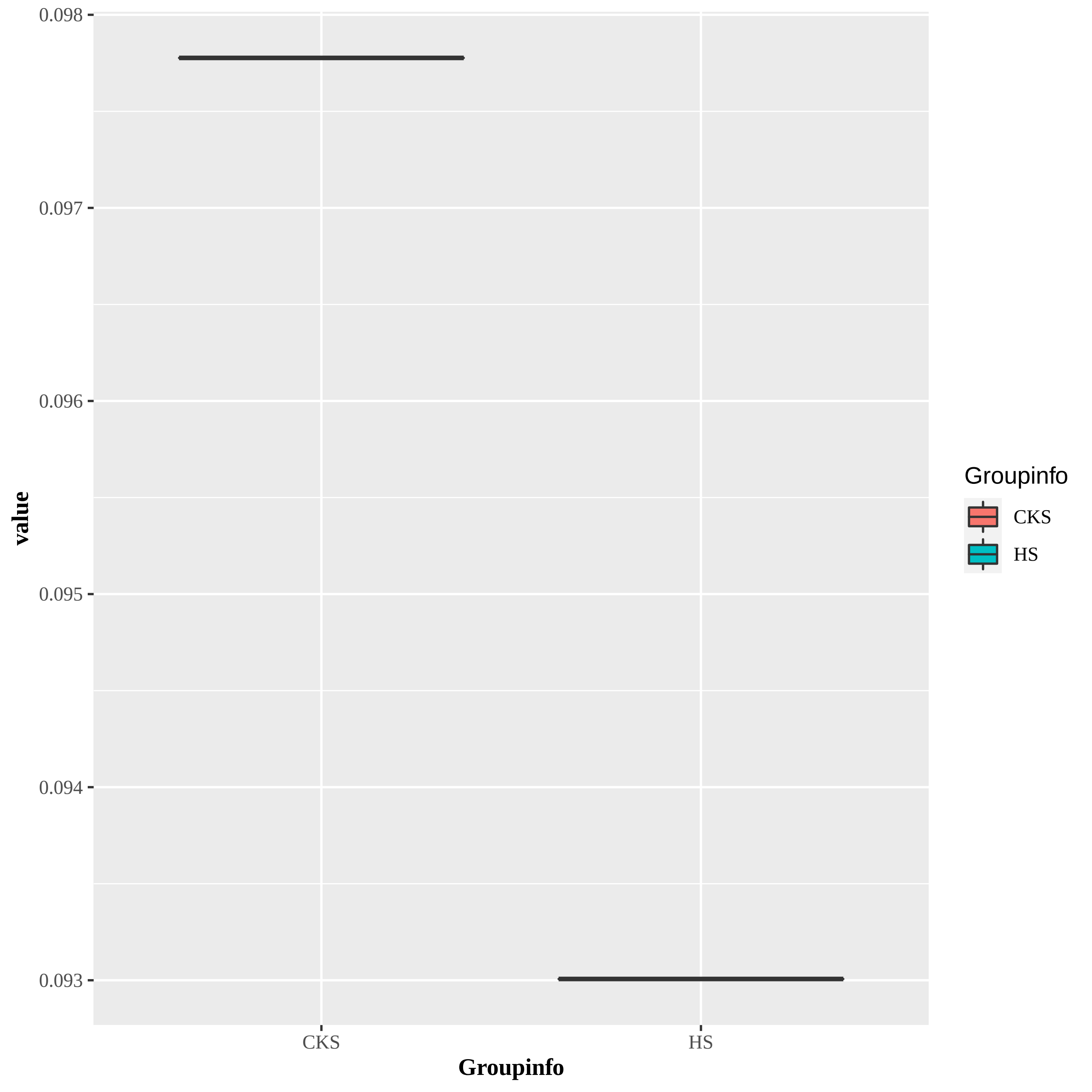
0.098
0.097
0.096
0.095
0.094
0.093
value
CKS
HS
Groupinfo
Groupinfo
CKS
HS
